# RETRACTION: Tanshinone sensitized the antitumor effects of irradiation on laryngeal cancer via JNK pathway

**DOI:** 10.1002/cam4.72040

**Published:** 2026-06-12

## Abstract

**RETRACTION**: XuH. , HaoY.‐L. , XuL.‐N. , ChenL. and XuF.‐W. , “Tanshinone sensitized the antitumor effects of irradiation on laryngeal cancer via JNK pathway,” Cancer Medicine 7, no. 10 (2018): 5187‐5193, 10.1002/cam4.1781 30239172 PMC6198231.

The above article, published online on 21 September 2018 in Wiley Online Library (wileyonlinelibrary.com), has been retracted by agreement between the journal Editor‐in‐Chief, Stephen Tait; and John Wiley & Sons Ltd. The retraction has been agreed following concerns raised by a third party. An investigation identified several instances of image duplication. Specifically, in Figure 4A, parts of the PBS panel were to be found duplicated in another article published later by a different author group. In addition, some western blot bands presented in Figure 5A were found to be duplicated in five other articles, three of which were published prior to this article. In one instance, the duplicated bands were used to represent different proteins. The authors were contacted to comment on the concerns and provide supporting data, but no response was received. The editors therefore consider the results and conclusions of this article to be compromised. The authors did not respond to our notice of retraction.

**RETRACTION**: H. Xu , Y.‐L. Hao , L.‐N. Xu , L. Chen and F.‐W. Xu , “Tanshinone sensitized the antitumor effects of irradiation on laryngeal cancer via JNK pathway,” Cancer Medicine 7, no. 10 (2018): 5187‐5193, 10.1002/cam4.1781 30239172 PMC6198231.

The above article, published online on 21 September 2018 in Wiley Online Library (wileyonlinelibrary.com), has been retracted by agreement between the journal Editor‐in‐Chief, Stephen Tait; and John Wiley & Sons Ltd. The retraction has been agreed following concerns raised by a third party. An investigation identified several instances of image duplication. Specifically, in Figure 4A, parts of the PBS panel were to be found duplicated in another article published later by a different author group. In addition, some western blot bands presented in Figure 5A were found to be duplicated in five other articles, three of which were published prior to this article. In one instance, the duplicated bands were used to represent different proteins. The authors were contacted to comment on the concerns and provide supporting data, but no response was received. The editors therefore consider the results and conclusions of this article to be compromised. The authors did not respond to our notice of retraction.

